# Polyethylene Stresses in Lumbar Total Joint Replacement Under Elevated Loading: Insights from an Anatomic Finite Element Model

**DOI:** 10.3390/bioengineering13010066

**Published:** 2026-01-07

**Authors:** Steven A. Rundell, Hannah Spece, Ronald V. Yarbrough, Steven M. Kurtz

**Affiliations:** 1 Explico Inc., Novi, MI 48375, USA; 2 School of Biomedical Engineering, Science and Health Systems, Drexel University, Philadelphia, PA 19104, USA; 3 Gyroid LLC., Haddonfield, NJ 08033, USA; 4 3Spine Inc., Chattanooga, TN 37402, USA

**Keywords:** spine, arthroplasty, elevated loading, finite element model, highly crosslinked polyethylene, wear, contact pressure, von Mises stress

## Abstract

The goal of this study was to assess elevated spinal loading conditions and their effect on the polyethylene stresses of a lumbar total joint replacement (L-TJR). A previously validated lumbar spine finite element model was virtually implanted with an L-TJR at L4–L5 and exposed to three elevated loading conditions: (1) 95th-percentile male body weight while bending forward, (2) combined ±7.5 Nm axial torsion and lateral bending, and (3) ASTM F2423 aggressive loading (1850 N plus 10–12 Nm bending). Combined torsion and lateral bending were considered because these loads and moments may be coupled in demanding real-world scenarios. Across all conditions, contact at the bearing remained confined to the intended spherical surfaces, consistent with Mode I in vitro wear tests, with no evidence of impingement. Contact stresses and von Mises stresses were considered acceptable based on the simulated results of Mode IV impingement tests. Only in one scenario—95th-percentile male body weight with multiaxial torsion—did von Mises stress in the polyethylene slightly exceed the stresses associated with impingement (<5%). These findings are useful in establishing the upper biomechanical loading limits for the L-TJR design beyond the 50th-percentile loading levels employed by standard in vitro tests. Future validation efforts such as a comparison with retrieval analyses or clinical data will further strengthen the model’s applicability to current and future questions of interest and contexts of use. Additional work may expand the modeling framework to incorporate patient-specific anatomy, variable implant positioning conditions, and a broader range of physiological load scenarios.

## 1. Introduction

Intractable low back pain represents a major unresolved health care and socioeconomic problem that has historically been treated by fusing the degenerated spinal levels [1,2,3,4]. Unfortunately, spinal fusion has been associated with adjacent segment degeneration [5,6,7]. During the past two decades, surgical interventions, such as lumbar total disc replacement (L-TDR), were developed to preserve the motion of degenerated spinal segments in an effort to prevent adjacent segment disease [8,9].

The MOTUS^®^ (3Spine Inc., Chattanooga, TN, USA) lumbar total joint replacement (L-TJR) is a motion-preserving spinal implant that replaces the function of the intervertebral disc and facet joints [10,11] and is currently undergoing a clinical trial in the United States via an Investigational Device Exemption (IDE). It is implanted via a posterior approach, similar to a bilateral transforaminal lumbar interbody fusion (TLIF) or posterior lumbar interbody fusion (PLIF) [10,11]. The L-TJR procedure is indicated for a single spinal motion segment following decompression at one lumbar level due to symptomatic lumbar degeneration with no more than Grade I spondylolisthesis [12,13]. In contrast, L-TDR is strictly indicated for degenerative disc disease and is placed using an anterior approach [14]. L-TDR is intended to share the load with the facets, which may be problematic if not fully biomechanically compatible or if the facet joints degenerate [15,16,17]. These limitations, coupled with difficulties in reimbursement, have diminished the popularity of L-TDR in the clinical community [14,18], resulting in an unmet clinical need for motion preservation in the lumbar spine that the MOTUS^®^ L-TJR is intended to address. Early clinical results for this device have shown encouraging outcomes with regard to decreases in back impairment and pain [19].

L-TJR achieves its biomechanical design objectives using two bilateral ultra-congruent spherical bearings, which are implanted along the pedicular axes [20]. The two bearings are fabricated from a combination of CoCr alloy components articulating against highly crosslinked, vitamin-E-stabilized ultra-high-molecular-weight polyethylene (hereafter, polyethylene) components. The bilateral spherical bearings allow flexion–extension but constrain axial torsion and lateral bending and resist horizontal shear. Because the bilateral spherical bearings are intended to withstand the lumbar segmental forces and moments that ordinarily would be resisted by the disc and facets, the mechanical robustness and resistance to wear of the design are of considerable clinical significance and interest to patients, surgeons, and regulatory agencies.

As part of the preclinical testing plan, the L-TJR was subjected to a battery of in vitro testing to assess the wear resistance under clean lubricant conditions (Mode I) as well as its resistance to abrasive wear (Mode III) and impingement (Mode IV) [20]. Overall, the L-TJR was found to exhibit the same or lower wear than previous L-TDRs. However, it must be emphasized that the standardized in vitro test methods were based on the clinical history, loading conditions, and wear performance of L-TDR. An equivalent volume of clinical history does not yet exist for L-TJR.

Consequently, we have developed and validated, via ASME V&V 40, a finite element model (FEM) of the L-TJR under established in vitro wear test conditions, so we could quantify the polyethylene stresses [21,22]. This characterization of the resulting stresses provides benchmarks for the subsequent in silico parametric analyses of component misalignment [22]. Additional assessments using this FEM have shown that stresses remain below that benchmark even under elevated loading conditions, at least in the context of a spine wear simulator [21]. We have also developed and validated a finite element model of the lumbar spine implanted with the L-TJR to examine the sensitivity of the polyethylene stresses to a variety of non-standard boundary conditions [23]. Our initial in silico studies with the implanted lumbar spine FEM focused on the effect of reasonable worst-case misalignment of the L-TJR in the axial and coronal planes [23]. During these previous misalignment simulations [22,23], the L-TJR was loaded with the standard equivalent of 50th-percentile male body weight.

In reality, some of the patients enrolled in the MOTUS^®^ U.S. clinical trial exceed the 50th-percentile male body weight and may be engaged in more demanding activities than forward bending. Therefore, the objective of the current study was to determine how increased loading affects polyethylene stresses and, thereby, evaluate the potential for surface and internal damage of the L-TJR. We employed the previously validated L-TJR Implanted Spine FEM and considered multiple types of elevated loading conditions including 95th-percentile male body weight with combinations of flexion–extension, lateral bending, and axial rotation. We hypothesized that the resulting stresses experienced by the polyethylene would be generally lower than what was determined by the same model when exposed to impingement boundary conditions (Mode IV). Thus, this study was intended to help establish the limits of loading and multiaxial torsion that are associated with the stress benchmarks from previous impingement Mode IV wear testing [20].

## 2. Materials and Methods

### 2.1. FE Model Development and Validation

We employed our previously validated lumbar spine FEM, consisting of two lumbar motion segments spanning L3 to L5 [23]. Briefly, for development of the model, a combination of automated and semi-automated image segmentation techniques (Analyze version 12.0, AnalyzeDirect, Inc., Lenexa, KS, USA) were used to extract detailed surfaces corresponding to the major bony structures of L3–5. The surfaces for the discs were based on anatomic bony landmarks and were imported into the commercial finite element mesh generation program, ANSYS (ANSYS Version R15, Inc., Canonsburg, PA, USA). The geometrical model of L3–5 was discretized into a combination of tetrahedral elements for the bony structures and hexahedral elements for the intervertebral discs. The nucleus pulposus was created to account for approximately 40% of the total volume of the intervertebral disc [24]. The disc height, measured at the center of the endplates, was measured to be approximately 12 mm.

The lumbar spine vertebrae (L3, L4, and L5) were modeled as rigid bodies. The L3–4 and L4–5 discs were modeled as deformable solids. The seven ligaments in the model (supraspinatus, ligamentum flavum, intratransverse, intraspinous, facet capsule, and posterior and anterior longitudinal elements) were modeled as linear springs. Muscle activation was modeled using nearly rigid cable elements (10,000 N/mm), so that they could generate the necessary tensile force to limit flexion in the presence of vertical, and horizontally offset, body loading.

**Figure 1:**
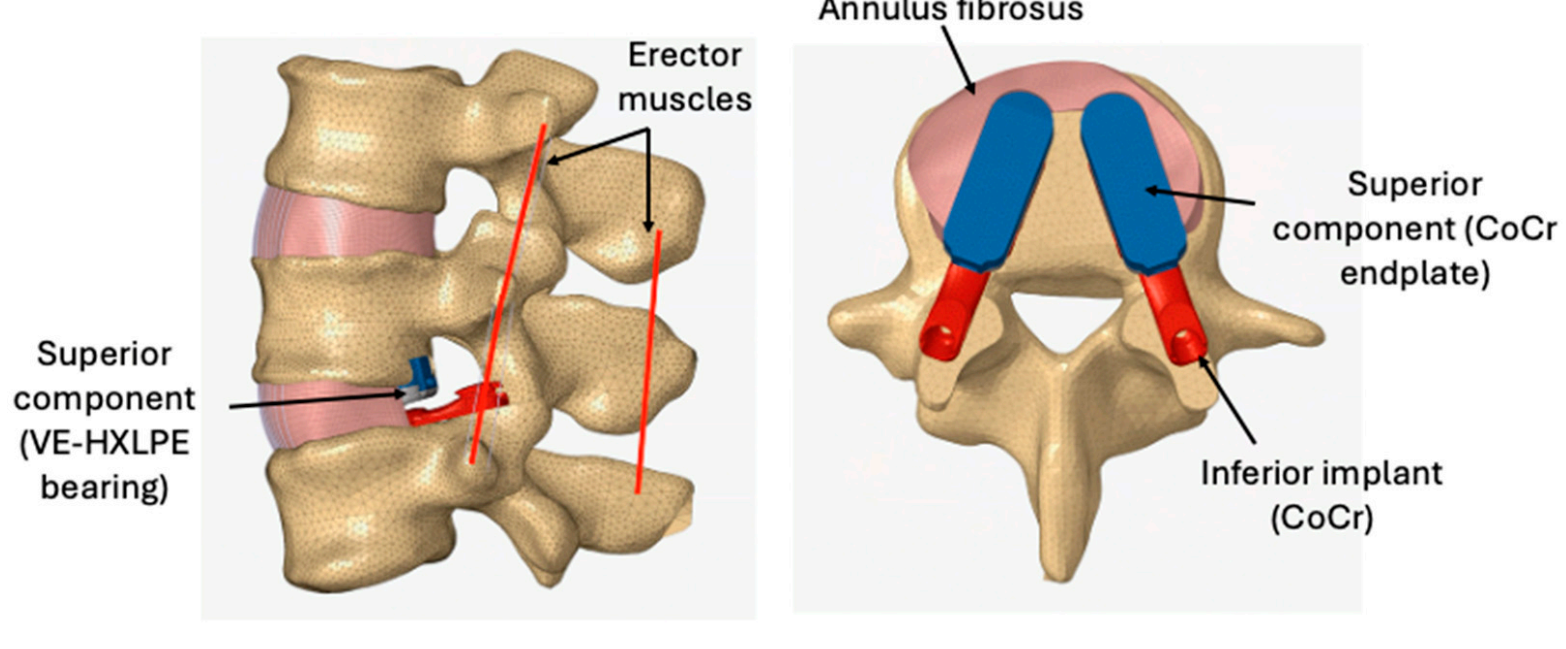
L-TJR implanted at L4–L5 in the lumbar spine FEM.

The previously validated FEM of the L-TJR device [21,22] was virtually implanted at L4–L5 in the lumbar spine FEM (Figure 1). The interface between the superior and inferior faces of the L-TJR components was modeled as tied contact and considered ideally fixed with no relative motion between the implant components and the bony endplates. The FEM maintained the anterior and lateral portions of the annulus fibrosus to reflect the specified surgical implantation technique. To reflect the excision of ligaments from a midline open surgical exposure during implantation, the posterior longitudinal ligament, the supraspinatus ligament, the intraspinous ligament, the ligamentum flavum, the facet capsular ligaments, and facets were removed from the FEM. We also removed the posterior erector muscle between the spinous processes of L4 and L5. Although the facet joints were visually present, the contact at those surfaces was computationally disabled. Based on surgeon input considering the dimensions of the spine FEM, a size 15 Long L-TJR was utilized.

For all analyses, we employed LS-DYNA in ANSYS’s explicit solver (Version R15). Explicit FEM is particularly suitable for problems involving high strain rates, large deformations, and complex contact interactions. Given the large amount of deformation likely to be experienced by the relatively soft discs, this method was determined to be suitable for the current modeling effort.

Our computational modeling approach has previously predicted real-world lumbar total disc replacement failures and informed in vitro wear testing [15,25]. For the present study, the lumbar spine FEM was formally verified and validated per ASME V&V 40:2018, as required by 2023 FDA guidance [26,27]. The overview and evidence of the L-TJR Implanted Spine FEM verification and validation activities have been described previously [23]. Briefly, verification efforts included code verification, mesh discretization optimization, confirmation of quasi-static performance, force balancing, and user error mitigation. Validation involved comparison of model outputs with in vitro and in vivo disc pressure and spine kinematics data and ensuring consistency between standalone and implanted L-TJR/spine models. The results of the V&V activities supported that the FEM is credible for its context of use.

### 2.2. Analysis of Elevated Loading Boundary Conditions Using the L-TJR Implanted Spine FEM

The primary Question of Interest (QOI) that the current study was intended to address was as follows: Based on the polyethylene stresses, is the L-TJR expected to be sufficiently durable under elevated loading boundary conditions? Previously performed physical wear testing for both Mode I and Mode IV wear was used to establish sufficient resistance to wear. The computational models were used to simulate these physical wear tests in order to establish a relationship between the computationally derived stresses and physical wear performance. In the current study, the computational model was used to evaluate the sensitivity of the L-TJR design to elevated loading with respect to a patient’s body weight and application of off-axis moments during bending in flexion. Specifically, the two types of elevated loading conditions examined by this study include reasonable worst-case loading (95th-percentile male body weight) and aggressive loading (bending in combination with axial rotation and multiaxial torsion) conditions to better understand and challenge the mechanical limits of the design. These aggressive loading conditions are not intended to replicate a patient’s activities of daily living.

#### 2.2.1. 95th-Percentile Male

The previous analyses performed with the L-TJR Implanted Spine FEM were based on flexion bending boundary conditions for a 50th-percentile male body weight. The current sensitivity analysis used a body weight that accounts for a 95th-percentile male individual to represent the maximum reasonable worst-case body weight scenario for our sensitivity analyses (308 pounds, 74 inches, BMI = 39.6 kg/m^2^). The basis for our worst-case loading scenario was the Instructions for Use (IFU) for the MOTUS^®^ L-TJR device, which specifies that patients with a BMI greater than 40 kg/m^2^ are contraindicated. Consequently, the previous flexion bending boundary conditions that utilize an upper body weight of 343 N were increased to 549 N.

#### 2.2.2. Lateral Bending and Axial Rotation for a 50th-Percentile Male

Our previous investigations of component misalignment using the Lumbar Spine Implanted L-TJR FEM focused on boundary conditions for flexion bending. They did not include simultaneous components for axial rotation and lateral bending [23]. Therefore, in the present study, the current model was altered to include these modes of loading. Specifically, the boundary conditions for flexion bending remained the same apart from the addition of the following:

1. A pure lateral bending moment applied at the superior endplate of L3 with a magnitude of 7.5 Nm;
2. A pure axial rotation moment also applied at the superior endplate of L3 with a magnitude of 7.5 Nm.

All combinations of positive- and negative-direction lateral bending and axial rotation were considered, resulting in a total of 4 (2 × 2) simulations. These analyses were conducted for a 50th-percentile male’s body weight.

#### 2.2.3. Lateral Bending and Axial Rotation for a 95th-Percentile Male

The prior two analyses were combined into a single analysis. Specifically, the L-TJR Implanted Spine FEM was run at a flexion bending with an upper body load of 549 N with a combined 7.5 Nm in axial rotation and lateral bending in all four permutations of directions (four simulations).

#### 2.2.4. Flexion–Extension, Lateral Bending, and Axial Rotation Torques per ASTM F2423

We also investigated the applied uniaxial torques in flexion–extension, lateral bending, and axial rotation as boundary conditions based on ASTM F2423 (Table 1) [28]. We adopted an in silico approach for these boundary conditions using the Lumbar Spine Implanted L-TJR FEM instead of an in vitro test, because the load control methodology proposed in the guide is practically problematic. Even in the guide, these applied torques are offered as an “alternative” method for testing an intervertebral disc prosthesis.

### 2.3. Polyethylene Outcome Measures

We considered the contact stress, also known as contact pressure, between the polyethylene and CoCr component surfaces, as well as the von Mises stress as the primary outcome measures for the polyethylene. Von Mises stress is an effective or equivalent stress metric that quantifies the distortion in the polyethylene and is associated with yielding, plastic flow, and failure of the polymer. Both contact stress and von Mises stress have historically been associated with wear and surface damage in the literature for polyethylene components in total joint replacement [24,29,30,31,32,33].

Previous computational analyses by the authors have evaluated the L-TJR device under Mode I and Mode IV wear conditions [22]. Specifically, the FEM employed in the present study was subjected to boundary conditions representative of standardized physical wear testing for both Mode I and Mode IV scenarios. Consequently, the von Mises stresses and contact pressures predicted in the current analyses can be directly compared to those obtained under these established testing conditions. Because Mode IV represents a worst-case wear scenario, comparing the present elevated loading results against Mode IV outcomes provides a meaningful assessment of device robustness. If the stresses observed in the current simulations are generally lower or consistent with those documented for Mode IV, and given that the device demonstrated acceptable in vitro performance under Mode IV conditions, it can be inferred that the device will also perform reliably under the edge-case loading scenarios investigated here.

## 3. Results

### 3.1. 95th-Percentile Male

**Figure 2:**
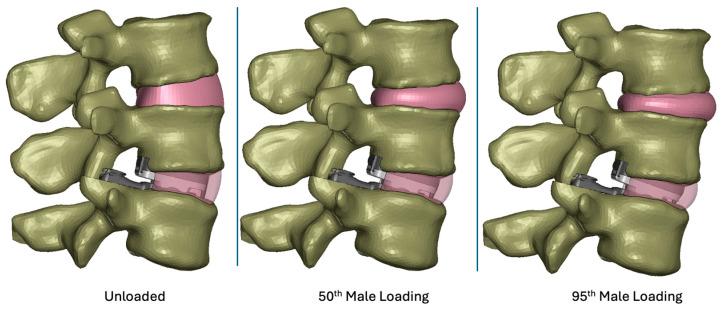
Images depicting the Implanted Spine FEM in an unloaded state (**left**), loaded consistent with a 50th-percentile male flexion bending (**middle**), and loaded consistent with a 95th-percentile male flexion bending (**right**).

**Figure 3:**
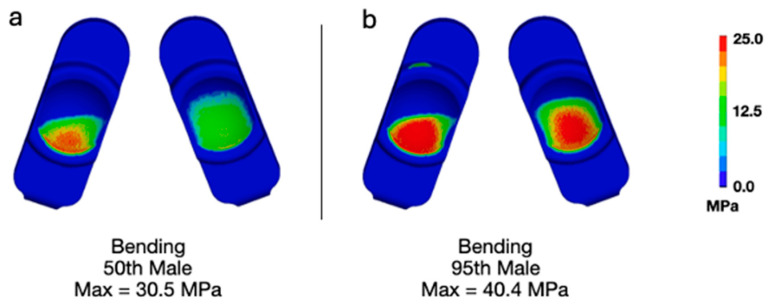
Contour plots of contact pressure during bending for (**a**) a 50th-percentile male and (**b**) a 95th-percentile male.

**Figure 4:**
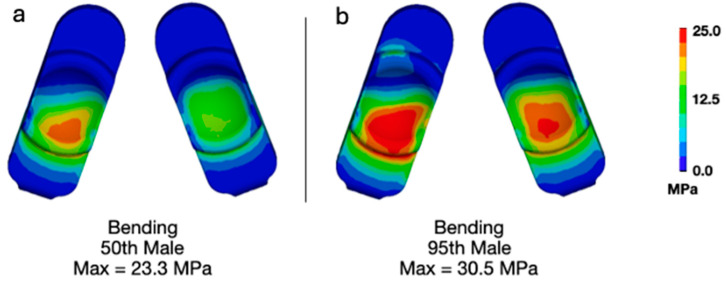
Contour plots of von Mises stress during bending for (**a**) a 50th-percentile male and (**b**) a 95th-percentile male.

Applying bending loads consistent with that of a 95th-percentile male generally increased the overall deformation of the model (Figure 2). Specifically, the increased loading added flexion and compression. Increasing the upper body weight from 343 N (50th-percentile male) to 549 N (95th-percentile male) generally increased the magnitudes of contact pressure and von Mises stress. Specifically, the maximum contact pressure increased from 30.5 (Figure 3a) to 40.4 MPa (Figure 3b). The maximum von Mises stress increased from 23.3 (Figure 4a) to 30.5 MPa (Figure 4b). The increased loading, which increased the overall level of flexion, caused contact to occur on the anterior aspect of the right side of the device (pictured as the left component in each set as devices are viewed from below). Regardless, the peak contact pressures and von Mises stresses remained lower than values previously determined to occur during impingement (Mode IV) boundary conditions (Peak Contact Pressure = 83.3 MPa and Peak von Mises Stress = 32.2 MPa).

### 3.2. Lateral Bending and Axial Rotation for a 50th-Percentile Male

**Figure 5:**
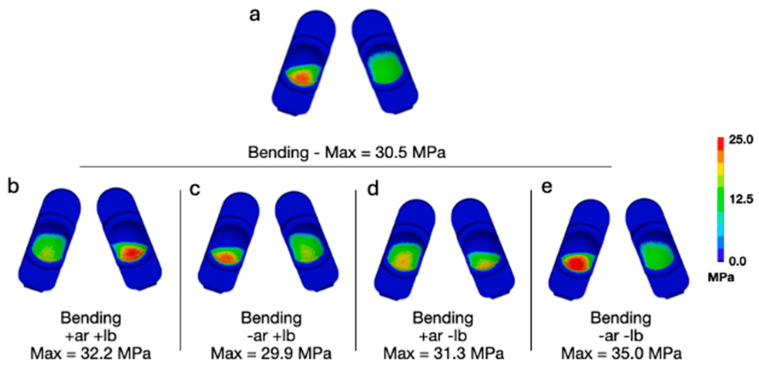
(**a**) Contours of contact pressure during flexion bending for a 50th-percentile male with no lateral bending or axial rotation moments applied, and (**b**–**e**) all variations of positive and negative moments applied at a magnitude of 7.5 Nm.

**Figure 6:**
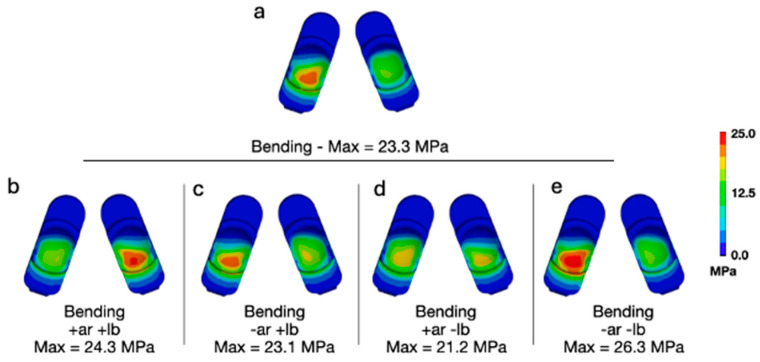
(**a**) Contours of von Mises stress during flexion bending for a 50th-percentile male with no lateral bending or axial rotation moments applied, and (**b**–**e**) all variations of positive and negative moments applied at a magnitude of 7.5 Nm.

Adding a combination of lateral bending and axial rotation while flexion bending generally increased the peak contact pressures and von Mises stress (Figure 5 and Figure 6). The L-TJR Implanted Spine FEM, during flexion bending with no lateral bending or axial rotation (Figure 5a and Figure 6a), exhibited a biased loading to the anatomical right (left component in each set) as well as posteriorly. Consequently, different combinations of axial rotation and lateral bending acted to redistribute the loading in a more symmetrical pattern. Specifically, during positive axial rotation and negative lateral bending (Figure 5d and Figure 6d), the contours of contact pressure and von Mises stress appeared the most symmetric, and, for von Mises, the peak value was reduced. The worst-case loading scenario appeared to be during a combination of negative lateral bending and negative axial rotation (Figure 5e and Figure 6e). The peak contact pressure and von Mises stress was 35 MPa and 26.3 MPa, respectively. The peak contact pressures and von Mises stresses remained lower than values previously determined to occur during impingement (Mode IV) boundary conditions (Peak Contact Pressure = 83.3 MPa and Peak von Mises Stress = 32.2 MPa).

### 3.3. Lateral Bending and Axial Rotation for a 95th-Percentile Male

**Figure 7:**
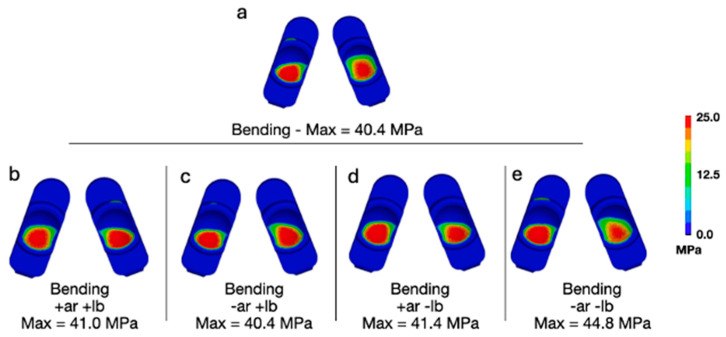
(**a**) Contours of contact pressure during flexion bending for a 95th-percentile male with no lateral bending or axial rotation moments applied, and (**b**–**e**) all variations of positive and negative moments applied at a magnitude of 7.5 Nm.

**Figure 8:**
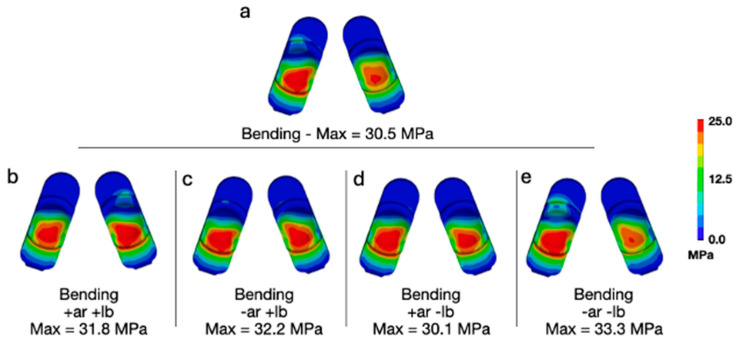
(**a**) Contours of von Mises stress during flexion bending for a 95th-percentile male with no lateral bending or axial rotation moments applied, and (**b**–**e**) all variations of positive and negative moments applied at a magnitude of 7.5 Nm.

Adding a combination of lateral bending and axial rotation while forward bending for a 95th-percentile male generally increased the peak contact pressures and von Mises stress when compared with flexion bending for a 50th-percentile male (Figure 7 and Figure 8). With respect to contact pressures, the addition of lateral bending and axial rotation to a 95th-percentile male bending scenario resulted in values between 40.4 and 44.8 MPa. These values are all substantially lower than those determined to occur during Mode IV impingement (83.3 MPa). For von Mises stress, the peak values during a 95th-percentile male bending scenario and various combinations of lateral bending and axial rotation ranged from 30.1 to 33.3 MPa. The peak von Mises stress determined to occur during Mode IV impingement was 32.2 MPa. Consequently, the stresses occurring during a 95th-percentile male bending scenario with lateral bending and axial rotation are slightly greater than that of Mode IV impingement. Notably, this phenomenon did not occur for contact pressure, which is governed by normal compressive loading and is far less affected by the multiaxial deformation generated under combined axial rotation and lateral bending compared to von Mises stress.

### 3.4. Flexion–Extension, Lateral Bending, and Axial Rotation Torques per ASTM F2423

**Figure 9:**
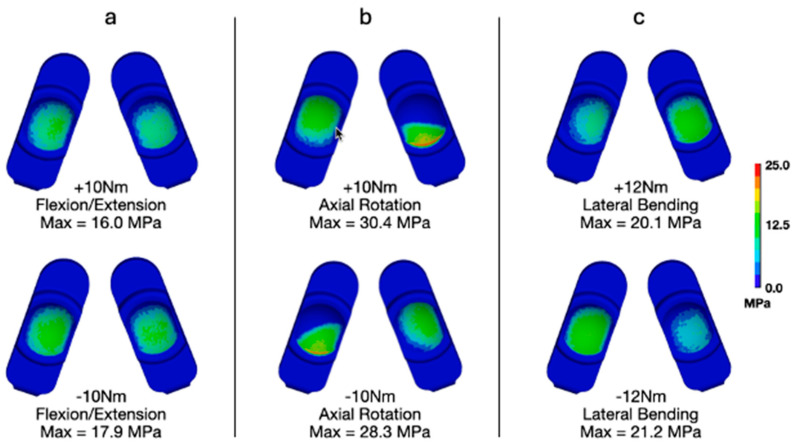
Contours of contact pressure during worst-case loading for (**a**) flexion–extension, (**b**) axial rotation, and (**c**) lateral bending.

**Figure 10:**
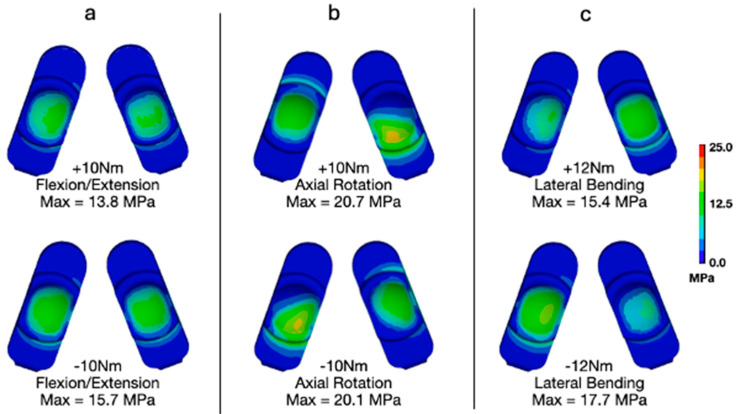
Contours of von Mises stress during worst-case loading for (**a**) flexion–extension, (**b**) axial rotation, and (**c**) lateral bending.

We also investigated the applied uniaxial torques in flexion–extension, lateral bending, and axial rotation as suggested in ASTM F2423 using a worst-case axial load of 1850 N (Figure 9 and Figure 10). With respect to contact pressures, applying the worst-case axial load resulted in values between 16.0 and 17.9 MPa, 30.4 and 28.3 MPa, and 20.1 and 21.2 MPa for flexion–extension (Figure 9a), axial rotation (Figure 9b), and lateral bending (Figure 9c), respectively. These values are all substantially lower than those determined to occur during Mode IV impingement (83.3 MPa). Similarly, for von Mises stress, the worst-case axial loading combined with flexion–extension (Figure 10a), axial rotation (Figure 10b), and lateral bending (Figure 10c) resulted in values between 13.8 and 15.7 MPa, 20.7 and 20.1 MPa, and 15.4 and 17.7 MPa, respectively. These values are also much lower than the peak von Mises stress determined during the Mode IV impingement scenario (32.2 MPa).

## 4. Discussion

In the present study, we challenged a novel L-TJR design using elevated loading. We adopted an in silico approach to address elevated loading, because standard preclinical tests are only based on normal body weight and the anticipated biomechanics for lumbar total disc replacement. Previous biomechanical studies of lumbar disc replacement have considered conceptual frameworks [34] or finite element modeling [15] to understand the effect of anterior constraint or device positioning on the loading of the facets. Whereas a total disc replacement shares the load with the facets, the L-TJR must be sufficiently robust to withstand the forces, moments, and motions across all three columns of the lumbar spine. In the absence of standards for the elevated loading of motion-preserving spine implants, we considered three types of elevated loading scenarios: 95th-percentile male body weight while flexion bending; a combination of ±7.5 Nm pure lateral bending moment and a ±7.5 Nm pure axial rotation moment; and loading conditions recommended by ASTM F2423. These combinations of forward bending and multiaxial torsion are considered an aggressive worst-case challenge for the L-TJR design. In one scenario, combining 95th-percentile loading, axial torque, and lateral bending moments, the von Mises stresses in the polyethylene were 3% greater than the impingement benchmark (32.2 MPa). It is unlikely that the aggressive multiaxial loading conditions considered here replicates any activity of daily living except, perhaps, yoga or vigorous snow shoveling by a 95th-percentile-male-body-weight patient (BMI > 40). The aggressive combined loading scenarios were considered to help characterize the limits of the L-TJR design. Overall, we found that, while elevated loading increased the stresses in the polyethylene, contact stresses generally remained below the levels associated with impingement benchmarked in a previous study.

Evaluation of the L-TJR Implanted Spine FEM indicated that the total axial force resisted by the components, when adding a combination of axial rotation and lateral bending, was approximately 4000 N (Table 2). The National Institute for Occupational Safety and Health (NIOSH) suggests that activities resulting in spinal compression should be less than approximately 3400 N for occupational lifting tasks [35]. Thus, the total axial force experienced by the construct during the current evaluation exceeded this suggested limit by approximately 600 N. The current results indicate that the construct is capable of withstanding exceptionally high axial forces. However, repeated exposure to such loading is not recommended, just as it would not be advisable for a native disc.

Little is known about how elevated loading would challenge the polyethylene bearings in this novel design. We validated our model of the L-TJR by simulating the in vitro wear and impingement tests under 50th-percentile [22] and 95th-percentile [22] body weight loading conditions, to ensure the FEM was credible for aggressive loading scenarios. We derived our contact stress and von Mises stress benchmarks from our prior simulations of in vitro wear tests, because those previous experimental studies showed that the wear and impingement behavior was comparable to or less than previously published lumbar total disc results. Thus, the wear testing of the L-TJR supported the reasonable safety profile for the design, when compared with the wear performance of other metal-on-polyethylene motion-preserving spinal implant designs that were approved by the FDA for clinical use in the USA.

Bartel’s work in the 1980s provided insight into the complex distribution of stresses in polyethylene components [29,31]. In the center of contact, the polyethylene is compressed at the surface, but the stresses are highly localized, and the unstressed regions around the contact zone act as a constraint, preventing lateral bulging or outward expansion like in a pure compression test. Rather than a uniaxial compression test, polyethylene contact is analogous to an indentation problem, in which the metal component indents the polyethylene. At the surface, the contact pressure is purely compressive, but, immediately below the surface, the constraining surrounding material gives rise to elevated hydrostatic stresses to support the loads at the bearing surface. The rate at which these hydrostatic stresses decay is a function of conformity of the bearing, and, as the hydrostatic stresses decrease, there is an opportunity for the material to distort, shear, and flow. Thus, even with a very simple linear elastic material model for the polyethylene, Bartel was able to elucidate that not only contact stresses, but also subsurface shear stresses, are relevant to consider in the potential generation of surface damage.

In the 1990s, with the incorporation of large deformation isotropic plasticity to more accurately model the stresses in polyethylene, the theories explaining the origins of subsurface damage shifted from considerations of maximum shear stress to von Mises yielding theory to define subsurface distortion [24]. Even by incorporating the yielding of the polyethylene, contact stresses for total knee replacements were calculated to be much greater than the yield stress of the polymer, which was counterintuitive and disturbing. Bartel et al. [24] explained the dichotomy as follows:

*“…polyethylene can withstand contact stresses that are considerably greater than the yield stress of the material. The hydrostatic component of stress, which is substantial for these designs, is not associated with damage. The damage is caused by distortion of the material as reflected in the von Mises stresses and strains. Consequently, it is not possible to set an absolute upper bound of contact stress on the basis of yield strength of the material.”*

For this reason, when designing a new total knee design for the Hospital for Special Surgery, the approach by Bartel and colleagues in 1995 was not to establish a numerical limit for acceptable stresses and strains for the design [24]. Instead, the proposed new knee design was benchmarked by analyzing contact stress and von Mises stress as performance metrics for the polyethylene.

Because total knee designs were available and on the market by the 1990s, Bartel was able to use the stresses and strains in historical designs to effectively benchmark the new design. Again, FEA was used as a tool to assess relative risk due to elevated stresses and strains, not absolute safety. We employed FEA of L-TJR in a similar manner. We use isotropic plasticity theory for highly crosslinked, vitamin-E-stabilized ultra-high-molecular-weight polyethylene (VE-HXLPE) to calculate the three-dimensional stresses in the polyethylene of the L-TJR. This material model is established in the literature for UHMWPE [24], and has several assumptions, namely, that the material hardens equally in all directions and does not depend upon the rate at which strain is applied. The model also assumes that the polymer is incompressible during yielding, which means that hydrostatic pressure has no effect on the yielding behavior. Instead, in this material model, yielding is based on the von Mises stress as a function of plastic strain. In other words, the 3% increased von Mises stress encountered in one of the aggressive combined scenarios was associated with the elevated yielding and plastic flow of the polymer and does not imply an increased failure risk for the VE-HXLPE.

Our analysis has limitations. First, the scope of the study was limited to reasonable worst-case and aggressive loading boundary conditions under the assumption of a baseline surgical alignment of the implant components. Second, we did not consider the combination of aggressive loading with surgical misalignment, nor did we consider aggressive loading associated with unreasonable misuse or revision scenarios such as subsidence or gross migration. Third, the model was limited to replicating the boundary conditions of L4–L5, corresponding to the most frequently studied lumbar levels examined in the literature, and did not address other levels of the spine, such as L5–S1, which would require the development, verification, and validation of an entirely different anatomical lumbar spine FEM. Fourth, the L4–L5 model in the present study was not developed or intended to be used to investigate patient-specific outcomes, which may necessitate replicating patient-specific anatomy, which was beyond the scope of our current investigation and would require additional validation activities. Overall, the limitations of the validated L-TJR Implanted Spine FEM do not diminish the credibility of the study presented here, and the ability of the analysis to rigorously address the QOI. Rather, the limitations identified here are intended to caution the reader about the potential generalization of our findings to other QOIs or COUs without undertaking the appropriate validation activities. Future work may expand the modeling framework to incorporate patient-specific anatomy, variable implant positioning conditions, and a broader range of physiological load scenarios. Additional validation efforts such as comparison with retrieval analyses or clinical data will further strengthen the model’s applicability to current and future QOIs and COUs.

## 5. Conclusions

In summary, we employed our previously validated L-TJR Implanted Spine FEM to investigate three elevated loading scenarios. Contact stresses and von Mises stresses were generally below those considered acceptable based on the simulated results of Mode IV impingement tests. Only in one scenario—95th-percentile male body weight with multiaxial torsion—did von Mises stress in the polyethylene slightly exceed the stresses associated with impingement (<5%). These findings are useful in establishing the upper biomechanical loading limits for the L-TJR design beyond the 50th-percentile loading levels employed by standard in vitro tests.

## Figures and Tables

**Table 1:** Summary of FEM runs to investigate ASTM F2423 boundary conditions.

ASTM Run	Maximum Load	Applied Torque
1	1850 N	+10 Nm Flexion/Extension
2	1850 N	−10 Nm Flexion/Extension
3	1850 N	+10 Nm Axial Rotation
4	1850 N	−10 Nm Axial Rotation
5	1850 N	+12 Nm Lateral Bending
6	1850 N	−12 Nm Lateral Bending

**Table 2:** Resultant contact force at the bearing surfaces of the L-TJR device.

Scenario	Contact Resultant Left (N)	Contact Resultant Right (N)	Total Axial (N)
Bending	1719	1631	3350
Bending +ar +lb	1999	2064	4063
Bending −ar +lb	2089	1918	4007
Bending +ar −lb	1766	2237	4004
Bending −ar −lb	1846	2215	4060

## Data Availability

The original contributions presented in the study are included in the article, further inquiries can be directed to the corresponding author.
